# Influence of automated visual field testing on intraocular pressure

**DOI:** 10.1186/s12886-020-01622-7

**Published:** 2020-09-09

**Authors:** Samuel Bertaud, Elisabeth Skarbek Borowski, Rachid Abbas, Christophe Baudouin, Antoine Labbé

**Affiliations:** 1 Quinze-Vingts National Ophthalmology Hospital, INSERM-DGOS CIC 1423, IHU FOReSIGHT, 28 rue de Charenton, 75012 Paris, France; 2 Department of Biostatistics and Epidemiology, Gustave Roussy, Villejuif, France; INSERM U1018, CESP, Université Paris-Sud, Université Paris-Saclay, Villejuif, France; 3 Department of Ophthalmology, Ambroise Paré Hospital, AP-HP, IHU FOReSIGHT, Université de Versailles Saint-Quentin en Yvelines, Université Paris Saclay, Versailles, France; 4 Sorbonne Université, INSERM, CNRS, Institut de la Vision, IHU FOReSIGHT, Paris, France

**Keywords:** Glaucoma, VF testing, intraocular pressure

## Abstract

**Background:**

To evaluate the influence of automated visual field (VF) testing on intraocular pressure (IOP) in patients with ocular hypertension (OHT) or glaucoma.

**Methods:**

We conducted a prospective observational study in the glaucoma department at Quinze-Vingts National Ophthalmology Hospital in Paris. Ninety-five right eyes of 95 patients followed for glaucoma or OHT were included. IOP was measured three times using a Nidek NT-510 non-contact tonometer within a maximum of 5 min before and after VF testing. Sub analyses using logistic regression analysis were performed to evaluate the impact of gender, age, central corneal thickness (CCT), mean deviation (MD) of the VF, VF test duration and filtration surgery on IOP fluctuations.

**Results:**

There was no significant change in IOP after VF testing, with IOP’s 15.14 ± 4.00 mmHg before and 14.98 ± 3.33 mmHg after the VF (*P* = 0.4). The average change in IOP was 0.15 ± 1.82 mmHg. Using multivariate analysis, no effect of the VF test on IOP was found (global model fit R^2^ = 0.12), whether based on duration of the VF test (*P* = 0.18) or the MD (*P* = 0.7) after adjustment for age, gender, CCT and history of glaucoma surgery. Similarly, there was no significant difference within different types of glaucoma, including open-angle glaucoma *(P = 0.36),* chronic angle closure glaucoma (*P* = 0.85) and OHT (*P* = 0.42). The subgroup of patients with an IOP elevation ≥2 mmHg had a significantly higher VF test duration (*P* = 0.002).

**Conclusion:**

VF testing does not influence IOP as measured with a non-contact tonometer.

## Background

Visual field (VF) testing and intraocular pressure (IOP) measurement are essentials for the diagnosis, monitoring and treatment of glaucoma [1, 2]. IOP reduction is the only evidence-based treatment that has been shown to slow glaucoma progression [3], and VF is the gold standard test for evaluation of glaucoma functional loss and progression [2, 4]. It has been demonstrated that IOP in healthy and glaucomatous eyes can be influenced by several factors, including accommodation [5–10]. During glaucoma or OHT patients follow-up, for logistical and organizational reasons, the VF is most often performed before clinical evaluation and IOP measurement. In daily practice, some ophthalmologists believe that VF testing might have an effect on IOP, in the form of a transient elevation. This potential IOP change might have a direct influence on glaucoma management, as the clinical decision to adjust glaucoma management is often based on the IOP.

Some studies have already investigated these possible IOP changes following VF testing, but with discordant results. Recupero et al. [11] concluded that IOP varied significantly and tended to increase immediately after automated visual field examination in 49 patients (94 eyes) with primary open-angle glaucoma (POAG). Ni et al. [12] showed similar results in a retrospective study of 109 right eyes of patients with POAG. However, Rebolleda et al. [13]*,* in 52 eyes of 27 patients with POAG, Martin et al. [14], in 61 patients with POAG or OHT, and Lee et al. [15]*,* in 71 eyes of 45 patients, found no impact of VF testing on IOP. Most of these studies used both eyes of the same patients or were retrospective. Moreover, no clear mechanism explaining this IOP rise following VF testing has been found. Several hypotheses have been suggested, including the examination in a dark room inducing a persistent mydriasis or a psychic stress leading to a sympathetic response that transiently elevates IOP [9, 16–18]. Nevertheless, as VF testing and IOP measurement are crucial in glaucoma management, if there is a real influence of VF testing on IOP, our clinical practice should be modified by dissociating IOP measurement and VF testing into two different examinations. The goal of this study was therefore to clarify the effect of VF examinations on IOP.

## Methods

We performed a prospective observational study in the glaucoma department of Quinze-Vingts National Ophthalmology Hospital in Paris. All patients were informed of the purpose of the study, and their consent was obtained in accordance with the declaration of Helsinki and approved by our CPP-Ile-de-France Ethical Committee (number 10793). Patients selected for inclusion had been followed for over 1 year for glaucoma or OHT and were over 18 years of age. They were required to have undergone at least three VF tests prior to inclusion. Exclusion criteria were difficulty in IOP measurement and any corneal pathology that might influence non-contact IOP measurement.

All VF tests and IOP measurements were performed by the same examiner with the same devices. VF analysis was performed with the Humphrey visual field analyser (HFA) 24–2 or 10–2 Swedish Interactive Threshold Algorithm standard program, or the OCTOPUS 101 G2 program and C08 program, or the Frequency doubling technology (FDT) matrix 24–2 pattern on undilated eyes. VF testing always started with the right eye. All VF tests were performed in the same department and under the same condition of illumination in a dark room. During the Humphrey and Octopus visual field tests, the near prescription lens was set as needed, and the fellow eye was occluded with a black patch. For the FDT matrix, the patients were measured with their distance correction if the spherical equivalent was outside of the range from − 3.00 to + 3.00 and no correction if inside this range. We ensured that patients were seated comfortably and perfectly understood the test. Mean deviation (MD) of HFA 24–2 or Octopus G2 were used for statistical analysis when patients had HFA 24–2 + HFA 10–2, Octopus G2 + Octopus C08 or HFA 24–2 + FDT performed during the same consultation. If patient had only a HFA 10–2 performed, the MD of this VF was used. The MD of FDT matrix was not used for MD analysis. IOP was measured within a maximum of 5 min before and after VF testing by the same Nidek NT-510 non-contact tonometer (Nidek CO, Gamagori, Japan) which have a test-retest variability of 6.4% [19].

At each time point, 3 consecutive measurements were taken to obtain a mean IOP value. We selected measurements only of right eyes to minimize intraindividual variations.

The change in IOP from immediately before to immediately after the visual field test was analysed with the paired t-test to account for intra-patient correlation. Multivariate linear mixed models were used to analyse IOP change adjusted for age, gender, central corneal thickness (CCT), visual field test duration, MD of visual field, and history of glaucoma surgery. Sub analyses of patients with IOP elevation or decrease ≥2 mmHg was done as it was in most studies concerning IOP variation after VF test [11, 13, 14].

## Results

**Fig. 1:**
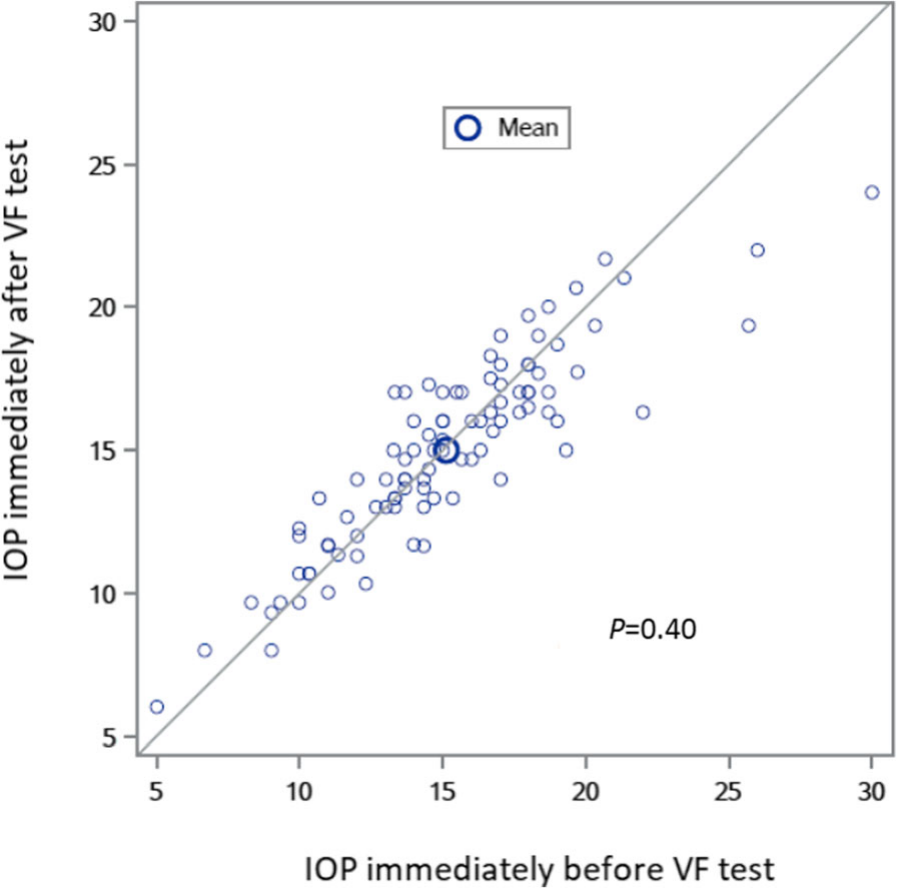
Agreement of IOP immediately before and immediately after VF testing. 1: IOP indicates intraocular pressure; VF visual field

One-hundred and two patients were examined, and 95 right eyes were included based on the predetermined criteria, with a mean age of 67 ± 12 years (range: 27 to 91). Baseline characteristics are presented in Table 1. There was no significant IOP change, with IOP 15.14 ± 4.00 mmHg before and 14.98 ± 3.33 mmHg after VF testing (*P* = 0.4, paired t-test) (Fig. 1). The mean IOP variation was 0.15 ± 1.82 mmHg, ranging from − 3.67 to 6.34 mmHg. A ≥ 2 mmHg difference in IOP before and after VF examination was observed in 23 eyes (24.2%), with a decrease in 13 eyes (13.7%) and an increase in 10 eyes (10.5%). The group with an IOP decrease ≥2 mmHg had a mean age of 59 ± 15 years, a mean VF test duration of 9,6 ± 2.9 min, a mean MD of − 5.8 ± 8.6 dB and a mean CCT of 552 ± 35 μm. There was no significant difference in patient characteristics between those having an IOP decrease ≥2 mmHg compared to patients without a ≥ 2 mmHg decrease (Table 2). The group with an IOP elevation ≥2 mmHg had a mean age of 64 ± 15 years, a mean VF test duration of 12,8 ± 2.6 min, a mean MD of − 9.8 ± 8.3 dB and a mean CCT of 537 ± 37 μm. Patients with an IOP elevation ≥2 mmHg had a statistically significantly longer VF test duration than other patients (12.8 ± 2.6 min. vs. 9.7 ± 3,0 min. Respectively, *P* = 0.002 (Table 3). Patients with an IOP elevation ≥2 mmHg had a statistically significant longer VF test duration as compared to patients with an IOP decrease ≥2 mmHg (12.8 ± 2.6 min. vs. 9,6 ± 2.9 min, respectively, *P* = 0,01) (Table 4). There was no significant difference for age, gender, CCT or MD between these two groups.

**Table 1:** Patient characteristics

Patients (*n* = 95)		
Age in years (mean ± SD)	67 ± 12	
Sex	Male 47 (49.5%)	Female 48 (50.5%)
Number of eyes using glaucoma medication	83 (87.4%)	
1 glaucoma medication	40 (42.1%)	
2 glaucoma medication	33 (34.7%)	
3 glaucoma medication	9 (9.5%)	
4 glaucoma medication	1 (1.1%)	
Glaucoma surgery (in right eyes)	12 (12.6%)	
Central corneal thickness (μm, mean ± SD, range)	544 ± 39 (439 to 648)	
VF test duration (min, mean ± SD, range)	9,6 ± 2,9 (4 to 17)	
Mean Deviation (dB, mean ± SD)	−5.8 ± 7.04	
Primary open angle glaucoma	74 (77.9%)	
Chronic angle closure glaucoma	13 (13.7%)	
Ocular hypertension	8 (8.4%)	
Types of VF test		
HFA 24–2 + 10–2	56 (58,9%)	
HFA 24–2	14 (14,7%)	
HFA 10–2	2 (2,1%)	
FDT + HFA 24–2	13 (13,7%)	
Octopus G2 + C08	8 (8,4%)	
Octopus G2	1 (1,1%)	
FDT	1 (1,1%)	

*VF* Visual field, *HFA* Humphrey Field Analyzer, *FDT* Frequency doubling technology

**Table 2:** Characteristics of patients with IOP decrease ≥2 mmHg compared with remainder of the patients

	IOP decrease ≥2 mmHg *N* = 13	No IOP decrease ≥2 mmHg *N* = 82	P
Age in years (mean ± SD)	58,.92 ± 14.84	67.76 ± 11.12	0.058^a^
Sex	Male 5 (39,5%) Female 8 (61,5%)	Male 42 (51,2%) Female 40 (48,8%)	0.58^b^
Glaucoma surgery (in right eyes)	1 (7,7%)	11 (13,4%)	1^c^
Central corneal thickness (μm, mean ± SD, range)	552 ± 35 (483 to 599)	542 ± 40 (439 to 648)	0.37^a^
VF test duration (min, mean ± SD, range)	9,6 ± 2.9	10,1 ± 3,1	0.58^a^
Mean Deviation (dB, mean ± SD)	−5.8 ± 8.63	−5.79 ± 6.9	0.81^a^

^a^Student’s t test

^b^Chi-squared test

^c^Fisher’s exact test

**Table 3:** Characteristics of patients with IOP elevation > 2 mmHg compared with remainder of the patients

	IOP elevation ≥2 mmHg *N* = 10	No IOP elevation ≥2 mmHg *N* = 85	P
Age in years (mean ± SD)	63.6 ± 14.7	66.9 ± 11.7	0.41^a^
Sex	Male 7 (70%) Female 3 (30%)	Male 40 (47%) Female 45 (53%)	0.20^b^
Glaucoma surgery (in right eyes)	3 (30%)	9 (10,6%)	0.11^b^
Central corneal thickness (μm, mean ± SD, range)	537 ± 37 (463 to 573)	545 ± 39 (439 to 648)	0.55^a^
VF test duration (min, mean ± SD, range)	12.8 ± 2.6	9.7 ± 3,0	***0.002***^***a***^
Mean Deviation (dB, mean ± SD)	−9.8 ± 8.3	− 5.35 ± 6.8	0.054^a^

^a^Student’s t test

^b^Fisher’s exact test

**Table 4:** Characteristics of patients with IOP decrease > 2 mmHg compared with patients with IOP elevation ≥2 mmHg

	IOP decrease ≥2 mmHg *N* = 13	IOP elevation ≥2 mmHg *N* = 10	P
Age in years (mean ± SD)	59 ± 15	64 ± 15	0.46
Sex	Male 5 (39,5%) Female 8 (61,5%)	Male 7 (70%) Female 3 (30%)	0.21^b^
Glaucoma surgery (in right eyes)	1 (7,7%)	3 (30%)	0,28^b^
Central corneal thickness (μm, mean ± SD, range)	552 ± 35 (483 to 599)	537 ± 37 (463 to 573)	0.32^a^
VF test duration (min, mean ± SD, range)	9,6 ± 2.9	12.8 ± 2.6	**0.01**^a^
Mean Deviation (dB, mean ± SD)	−5.8 ± 8.6	−9.8 ± 8.3	0.29^a^

^a^Student’s t test

^b^Fisher’s exact test

**Fig. 2:**
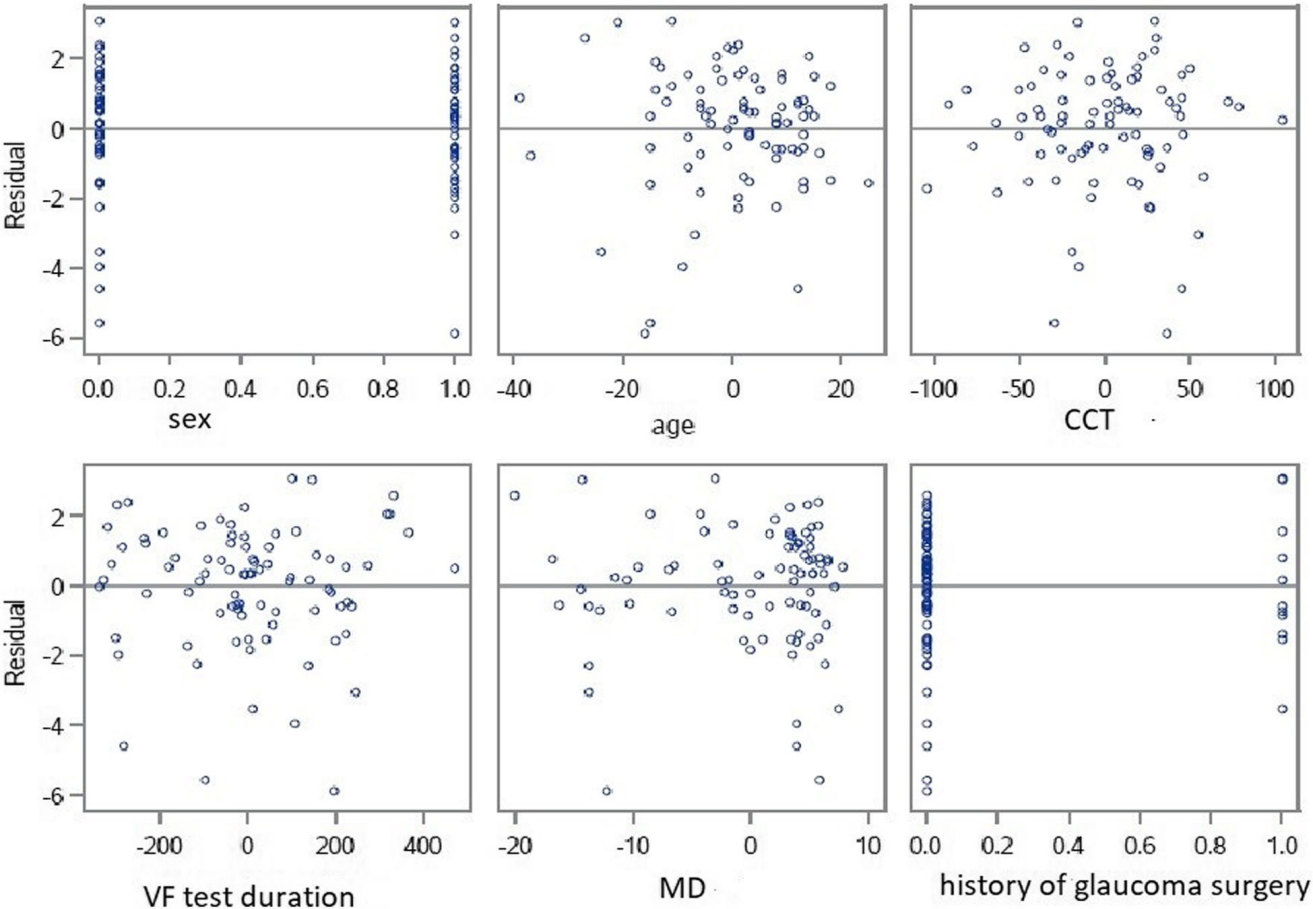
Multivariate analyses. Residual by regressors for IOP change (sex, age, CCT, VF test duration, MD, history of glaucoma surgery). IOP indicates intraocular pressure; CCT central corneal thickness, VF visual field; MD mean deviation

On multivariate analysis, no impact of the VF test on IOP was found (global model fit R^2^ = 0.12) whether based on the duration of the VF test (*P* = 0.18) or the MD (*P* = 0.7) after adjustment for age, gender, CCT and history of glaucoma surgery (Fig. 2). No difference was found in either the subgroup of patients with open angle glaucoma (*P* = 0.36) or the subgroups of patients with chronic angle closure glaucoma (*P* = 0.85) or OHT (*P* = 0.42).

## Discussion

As analysis of the VF is essential in the evaluation of glaucoma progression, and in order to avoid abnormal VF results related to previous examination of the eye, glaucoma patients often have the VF test performed just prior to the ophthalmological consultation. IOP is the only parameter amenable to treatment, with proven efficacy on glaucoma progression, so its measurement during the consultation has a direct impact on the therapeutic decision. However, it has been stated that VF testing might increase IOP in some patients. This could be of particular importance and may justify performing the VF test at a separate time from that of the consultation. It is well known that environmental factors have an effect on IOP. Erb et al. showed that psychological stress can induce an IOP elevation, possibly due to a sympathetic response increasing blood pressure and heart rate [16]. Patients who see the VF as a difficult performance test could be stressed, and this could be a factor causing an elevation in IOP after the VF test. VF’s are also performed in a dark room, and Gloster et al. showed that mydriasis induced by confinement in a dark room resulted in an IOP elevation of 4 mmHg (not only in angle closure glaucoma), with the return to the original IOP taking approximately 10 min [9]. In our study we found no impact of the VF test on IOP (global model fit R^2^ = 0.12), whether based on the duration of the VF test (*P* = 0.18) or the MD (*P* = 0.7) after adjustment for age, gender, CCT and history of glaucoma surgery, analysing all patients. Patients with a ≥ 2 mmHg IOP elevation had a mean VF test duration of 12.8 ± 2.6 min, which was a longer test duration than for other subjects (*P* = 0.002, Table 3), suggesting an effect of VF testing on IOP for patients with longer VF test durations. However, this was only found on a subgroup analysis, and there were no correlations observed on multivariate analysis between IOP change and VF test duration.

In the literature, several studies have already studied this subject and have led to conflicting results. Two studies showed an impact of VF testing on IOP. Recupero et al. reported a significant mean increase in IOP after automated VF testing (*P* < 0.01) in 94 glaucomatous eyes [11]. The mean IOP increase was 2.38 ± 3.49 mmHg (range - 6 to 11 mmHg). They also found that elderly glaucoma patients showed a significantly higher IOP elevations than younger patients, which was not confirmed in our study. They suggested rejecting IOP measurements in glaucomatous eyes if measured soon after VF testing. Interestingly, in this study, test duration ranged from 7 to 21 min, using the central 30–2 full-threshold program, which is typically a VF test with a longer duration. This longer duration of the VF test might be associated with psychological stress, inducing an IOP elevation [16, 20]. Similarly, our patients with a transient IOP elevation also had a significantly longer duration of tests. Another retrospective study of 109 eyes by Ni et al. [12] reported that the mean IOP measured after VF testing (14.9 ± 4.7 mmHg) was significantly higher than both the previous (13.7 ± 4.4 mmHg, *P* < 0.001) and following visits without VF examinations (13.8 ± 4.4 mmHg, *P* < 0.001). They explain the rise in IOP by a stressful perception of VF testing by the patients, leading to a sympathetic response that transiently elevates IOP [21]. Nevertheless, they did not find any correlation of IOP variation with age or use of β-blockers (BB) or α-2-agonists (AA). In this study, IOP measurements were not compared immediately before and after VF testing, but were compared to the previous visit and the following visit. Factors other than the VF test might have interfered with IOP and could explain these different results.

Conversely, three studies showed no differences. Rebolleda et al. [13] studied 52 eyes (27 patients) with POAG and did not observe any significant IOP variations immediately after VF testing or 1 h later. They did not find any significant IOP change in a group of 12 eyes (6 patients) who underwent Goldman tonometry immediately before and after VF testing. They used the same patients as a control group and repeated tonometry 72 h later, in the same time frame but without VF examinations, and they did not find any significant IOP variations either. They used a control group, because they believed that the IOP variations found by Recupero et al. [11] could be due to fluctuations in the IOP inherent to glaucomatous eyes and not to an effect of VF testing. Martin et al. [14] studied 61 patients with glaucoma, OHT or suspected glaucoma, measuring IOP immediately before and after VF testing, and the mean differences were not significant. Lee et al. [15] studied 45 patients (71 eyes) with OAG with the Icare® rebound tonometer (RBT). The IOP’s measured immediately and 20 min after VF testing were not different, and the IOP measured 10 min after VF testing was significantly lower (− 0.57 ± 1.84 mmHg) but within the margin error of the RBT. In the present study, IOP measurements were taken using a non-contact tonometer, which is not considered as the gold standard for tonometry measurement, as is Goldman applanation tonometry [22]. The reason was to avoid disturbing either the visual field or the second IOP measurement with anesthetics and/or repetitive procedures and manipulations of the eye and the patient. In addition, we excluded patients with pathological corneas or other conditions that could cause errors in IOP measurements, which might have affected the reliability of IOP measurement. Moreover, the two measurements before and after the VF were taken by the same method and on the same machine. This may have limited the issue of subjectivity in Goldmann tonometry and avoided measurement bias. Our study measurements were done within a maximum of 5 min before and after VF testing for every patients, allowing observation of a direct effect of VF testing if it existed. Intraocular pressure is a highly variable value, and it is difficult to analyse it with a single measurement as it is done in this study, it could be interesting to evaluate IOP change before and after VF test of the same patients in the same conditions several times to see if the same observations are found. In addition, no difference was found with regard to severity of glaucoma, history of glaucoma surgery, use of glaucoma medication or age. As usual in clinical practice, patients had different types of VF tests performed during the consultation, including HFA 24–2 alone or combine with 10–2, Octopus G2 alone or combine with Octopus C08, and FDT matrix 24–2. Nevertheless, only 2 patients had a HFA 10–2 alone (2,1%) and only one patient (1,1%) had a FDT alone and his MD value was not considered for statistical analysis. Although it would have been interesting to analyse the effect on IOP of the different types of VF, the low number of patients in each group did not allow this subgroup analysis in our study.

## Conclusion

Our study confirms that there is no effect of visual field testing on IOP. One may therefore continue to schedule visual fields just prior to the consultation in clinical practice when preferable, which facilitates the coordination of care for the doctor and patient and helps to ensure proper compliance with patient follow-up.

## Data Availability

The datasets during during the current study available from the corresponding author on reasonable request.

## Abbreviations

BB: β-blockers
CCT: Central corneal thickness
FDT: Frequency doubling technology
HFA: Humphrey field analyser
IOP: Intraocular pressure
MD: Mean deviation
OHT: Ocular hypertension
POAG: Primary open-angle glaucoma
RBT: Rebound tonometer
VF: Visual Field
